# Spatial mapping of Ethiopian cutaneous leishmaniasis lesions reveals distinct tissue level immune programs

**DOI:** 10.3389/fimmu.2026.1870524

**Published:** 2026-07-21

**Authors:** Nidhi S. Dey, Thao-Thy Pham, Shoumit Dey, Mekibib Kassa, Tigist Mekonnen, Helina Fikre, Pieter Monsieurs, Spatial CL Consortium, Myrthe Pareyn, Johan van Griensven, Malgorzata Domagalska, Jean-Claude Dujardin, Mezgebu Silamsaw Asres, Mikias Woldetensay, Paul M. Kaye, Wim Adriaensen

**Affiliations:** 1 York Biomedical Research Institute, Hull York Medical School, University of York, York, United Kingdom; 2 Department of Clinical Sciences, Institute of Tropical Medicine, Antwerp, Belgium; 3 Leishmaniasis Research and Treatment Centre, University of Gondar, Gondar, Ethiopia; 4 Department of Biomedical Sciences, Institute of Tropical Medicine, Antwerp, Belgium; 5 Department of Internal Medicine, University of Gondar, Gondar, Ethiopia; 6 Department of Dermatovenereology, University of Gondar, Gondar, Ethiopia

**Keywords:** cutaneous leishmaniasis, granulomas, immunopathology, skin, spatial transcriptomics, tertiary lymphoid structures, epithelial hyperplasia

## Abstract

**Introduction:**

Human cutaneous leishmaniasis (CL) is a prevalent but neglected tropical disease characterised by inflammatory lesions that are either restricted to the site of the infected sand fly bite or disseminate to mucosae or within the skin. Compared to well-studied pre-clinical models, the nature and diversity of the tissue response in humans remains to be fully appreciated.

**Methods:**

We conducted an exploratory study using spatial transcriptomics on paired lesional and non-lesional skin punch biopsies from five Ethiopian CL patients (two infected with *L. aethiopica*, one with *L. tropica*, and two with species unconfirmed). We used reference-free deconvolution, morphology-guided regional analyses, and immunohistochemistry-based validation to characterise the tissue responses.

**Results and Discussion:**

We identified spatially distinct immunopathological tissue responses including: i) epithelial hyperplasia with interferon-stimulated keratinocytes, ii) cytotoxicity with tertiary lymphoid structures, iii) granulomatous inflammation with proinflammatory response, iv) granulomatous inflammation with M2-polarised myeloid cell responses, and v) fibrotic remodelling with active collagen synthesis. Each patient in this case series exemplified one of these tissue responses, but immunopathological features were not mutually exclusive. This study extends our understanding of Ethiopian CL immunopathology and provides a molecular and cellular context that can be applied in larger clinical cohorts for testing hypotheses regarding the host and/or parasite determinants of CL disease diversity.

## Introduction

The cutaneous leishmaniases, caused by infection with several different species of *Leishmania*, are some of the most important neglected tropical diseases affecting the skin with approximately one million new cases annually, across more than 70 countries (1). For example, in the Ethiopian highlands, up to 29 million people are at risk with an estimated 20,000-50,000 annual cases (2), predominantly affecting young adults and children (3). Facial lesions represent 45 - 97.6% of Ethiopian cases (4) and active lesions or disfiguring scars can lead to stigma, social exclusion, and poor mental health (5). Current treatments remain inadequate, with 32-38% failure rates (6, 7) and treatment-related morbidity that further compromises quality of life (8).

In Ethiopia, *L. aethiopica* is the dominant causative agent of cutaneous leishmaniasis (CL) producing an unusually broad range of clinical presentations (9), from self-healing localized lesions to chronic disfiguring disease. CL due to *L. major*, *L. donovani* (9, 10) and *L. tropica* (11) infections has also been reported (12). Localized CL (LCL) is most prevalent, but mucosal and mucocutaneous disease is also frequent (7, 13), while diffuse cutaneous leishmaniasis (DCL) is rare and presents as non-ulcerating nodules (14–16). Clinical as well as preclinical studies in rodent and primate models of *L. major* and *L. amazonensis* LCL have indicated that protective immunity involves highly regulated Th1-polarized CD4^+^ T cell responses and nitric oxide and reactive oxygen species-producing macrophages (17). In contrast, exaggerated inflammatory responses observed in mucosal and disseminated American tegumentary disease caused by *L. braziliensis* are driven by cytolytic/senescent CD4^+^ and CD8^+^ T cells, NK cells and local environmental cues, including hypoxia and inflammasome activation (18–21). Conversely, immunosuppressive cytokines (IL-10, TGF-β) (22–24) and “anergy” are associated with DCL in *L. aethiopica* and *L. braziliensis* infections (25).

Despite being first reported in the 1930s (26, 27), the immunopathogenesis of Ethiopian CL remains poorly characterised at the tissue level. Histologically, Ethiopian CL lesions show predominantly dermal lymphohistiocytic inflammation with variable presence of granulomas (28), neutrophil-mediated tissue damage via death ligands (FasL, TRAIL) (29) and elevated arginase in lesions (30). A recent analysis of plasma chemokines and cytokines and parasite sequencing failed to identify systemic immune signatures or parasite genetic factors associated with different clinical presentations (31) suggesting that pathological mechanisms may be localized to lesional tissue. These studies have provided only a glimpse at the complexity underlying the immune response during human CL, limited by the number of analytes/cell types that are studied in any individual patient (32). More recently, bulk (20, 33–35) and spatial (36–38) transcriptomics have been used to provide a more holistic picture of the immune landscape associated with CL lesions, with the latter best suited to the identification of specialised niches within complex tissues. To date, similar studies have not been conducted in the context of CL in Ethiopia or elsewhere in East Africa.

In this exploratory study, we sought to characterise the immune response at the lesional level in patients presenting with CL in Ethiopia. We used spatial transcriptomics to map tissue responses in five individual patients, using paired lesional and non-lesional biopsies to distinguish disease-associated transcriptional programmes from baseline skin signatures. We explored heterogeneity between patients, and identified dominant immunopathological niches. These exploratory studies provide a framework for further research aimed at understanding the immunological and/or parasitological basis for disease heterogeneity in Ethiopia.

## Results

### Distinct transcriptomes in lesional and non-lesional skin of CL patients

**Figure 1:**
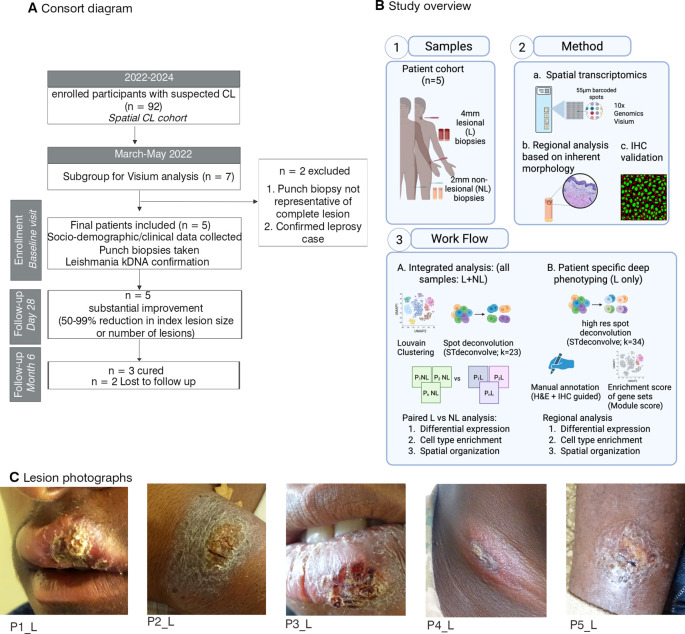
Study design and patient cohort characteristics. **(A)** CONSORT diagram showing patient recruitment, inclusion criteria, and final study cohort. **(B)** Schematic representation of study design showing patient cohort, types of biopsies collected, and analysis workflow of spatial transcriptomics data. **(C)** Lesion photographs of the 5 patients included in the study.

For this exploratory study, we recruited five male patients of homogenous demographic composition (P1-P5; median age 21 years, inter-quartile range [IQR]: 20-23) from diverse occupational and educational backgrounds from Gondar city in the Amhara Province, northern Ethiopia (**Supplementary Table 1**). Of seven patients initially enrolled, two were excluded prior to analysis (see Methods). All patients provided biopsies prior to treatment with sodium stibogluconate (SSG) and treatment response was assessed at one and six months follow up. P3 and P4 were lost to follow up after one month but treatment response for all patients ranged from 50-99% at day 28, as determined by reduction of lesion size, induration and number of active lesions (**Figure 1A**; **Supplementary Table 2**). A schema indicating the methods and workflow of the study is provided in **Figure 1B**. Lesion appearance (**Figure 1C**) was documented along with detailed clinical and laboratory findings for each patient at baseline. Common lesion characteristics included plaque formation (3/5 of all patients), scaling (4/5), crusting (5/5), swelling (5/5), and erythema (4/5), with some cases showing additional features such as hyperpigmentation (1/5) and signs of bacterial infection (2/5). *Leishmania* species were confirmed in three patients: two *L. aethiopica* (P1, P2) and one *L. tropica* (P5) (**Table 1**).

**Table 1:** Clinical and laboratory data at baseline visit.

PID^¥^	Duration of lesion in months	Number of active lesions	Lesion size index lesion (cm2)	Location of index lesion	Characterization of index lesion	Location of other lesions	Characterization of other lesions	Scars	other dermatological conditions	Parasite microscopy grade	kDNA PCR^#^ (Ct)	Parasite species typing^Δ^
P1	6	1	4	lip	crusted, macrochelial, swollen, erythematous, super-infected	NA	NA	No	acne vulgaris	4+	9.94	*L. aethiopica*
P2	26	1	15	arms	plaque, scaly, crusted, swollen, erythematous, hyperpigmented	NA	NA	No		0	27.31	*L. aethiopica*
P3	4	3	8	lip	plaque, scaly, crusted, dry, macrochelial, swollen, exfoliative, erythematous	arms, ear	nodular, scaly, crusted, dry, swollen, erythematous	No		0	33.42	Not done
P4	11	1	21	neck	nodular, scaly, crusted, dry, swollen, erythematous	NA	NA	No	acne vulgaris	0	29.03	Not done
P5	1	3	20	legs	plaque, scaly, crusted, dry, swollen, super-infected	ear, chest	papular, plaque, erythematous	No		0	20.03	*L. tropica*

^¥^ All patients had CL as primary infection with no previous history of CL lesions, PID: Patient identifier; ^#^kDNA PCR, kinetoplast DNA polymerase chain reaction

^Δ^verified either with SureSelect or high-resolution melt PCR or ITS-1 amplicon seq

**Figure 2:**
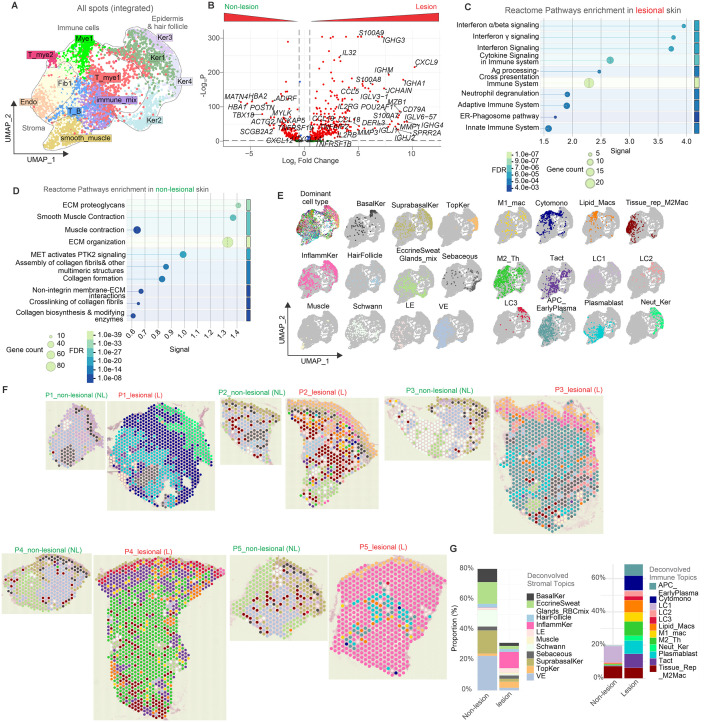
Global cellular heterogeneity and spatial organization in lesional vs non-lesional skin. **(A)** Uniform manifold approximation and projection (UMAP) of pooled non-lesional and lesional skin spots labelled by major skin domains inferred from gene expression. **(B)** Volcano plot of differentially expressed genes (UMAP) between non-lesional and lesional samples (red dots = adjusted P < 0.05, log2Fold > ± 0.25, -log10p > 10). **(C, D)** Top 10 Reactome pathways enriched in lesional **(C)** and non-lesional **(D)** skin. Bubble size = gene count; bars = signal strength (weighted harmonic mean of observed/expected ratio and -log (FDR); pathways grouped by similarity (Jaccard index ≥ 0.8, ranked by p-value). **(E)** UMAP coloured by dominant deconvoluted topic (highest proportion per spot) detected in each spot. **(F)** Spatial maps of non-lesional and lesional skin (P1-P5) coloured by dominant topic per spot. **(G)** Relative proportions of stromal (right) and immune (right) topic in non-lesional and lesional skin. All panels show pooled data from n=10 sections (5, non-lesional and 5 lesional from 5 patients) except **(F)**, which shows individual patient maps.

To compare the spatial organisation of immune and stromal cells between lesional and non-lesional skin, we obtained one lesional and one non-lesional biopsy at baseline (before the start of treatment) from each patient and performed spatial transcriptomics on the 10x Genomics Visium platform (**Figure 2**). Data were filtered to remove low-quality spots and lowly expressed genes; spots with fewer than 80 total counts and genes detected in fewer than 8 reads across all spots were excluded. A total of 5562 spots (1583 non-lesional and 3979 lesional) were retained and pooled for downstream analyses. The total number of genes per spot was consistently higher in lesional compared to non-lesional skin, reflecting the increased cellularity of lesional samples (**Supplementary Figure 1A**).

Unsupervised clustering of all samples using the Louvain algorithm (39) identified 12 distinct clusters based on gene expression (**Figure 2A**; **Supplementary Figure 1B**). Using cluster-specific gene expression signatures, we annotated three major spatial domains: i) epidermal and hair follicle, characterized by keratinocyte markers (in Ker1–4 clusters), ii) dermal immune enriched (immune_mix, T_B, T_mye1, T_mye2, Mye1 clusters) and iii) connective stromal tissue, rich in fibroblasts (fib1), smooth muscle cells (smooth_muscle cluster) and endothelial cells (Endo cluster). These annotations clearly separated in the UMAP visualization and spatial diagrams (**Supplementary Figures 1C, D**) despite the integrated analysis of non-lesional (NL) and lesional (L) samples. This suggests a preservation of core transcriptional programs defining major skin compartments even in the disease state.

We next conducted differential gene expression analysis between all lesional and non-lesional skin to identify differentially abundant transcripts (**Figure 2B**; Wilcoxon rank sum test; Bonferroni FDR correction). Lesional tissue showed marked upregulation of class switched immunoglobulin genes (*IGHA1*, *IGHG3*, *IGHG4*, *JCHAIN*), reorganisation of extracellular matrix (ECM; *MMP3*, *MMP1*), antimicrobial response (*SPRR2A*) and inflammatory markers (*S100A7*, *S100A8*, *CCL5*, *CXCL9*, *IL32*, *CCL18*, *CCL19*), while non-lesional skin was enriched for structural and stromal genes including *POSTN*, *MATN4*, *ADIRF* and *TBX18*. Gene set enrichment analysis (GSEA) revealed significant Reactome Pathway enrichment in lesional skin associated with interferon and other cytokine immune responses, antigen processing and presentation, neutrophil degranulation and ER (Endoplasmic Reticulum)-phagosome pathway (**Figure 2C**). In contrast, GSEA of non-lesional skin reflected the dominant stromal cell composition (**Figure 2D**).

Visium spots (55µm diameter) represents a tissue niche where 5–12 cells may be co-localised. To estimate which cell types were contributing to the changes in transcriptomes of lesional and non-lesional tissue, we used a reference-free approach (STdeconvolve) based on inherent gene expression profiles to identify the cell-type composition of each spot (40). Based on chosen resolution (see methods) STdeconvolve is able to generate gene expression profiles (termed “topics”) that are either lineage-enriched or mixed i.e., they contain genes associated with multiple lineages. Mixed topics thus represent either spatial co-occurrence of cell types or the presence of shared transcriptional programs active in multiple lineages. Using this deconvolution approach, we estimated topic proportions for each spot. At a coarse resolution (K = 23), we identified 11 stromal/epidermal and 12 immune cell topics, (see gene expression of topics shown in **Supplementary Figures 1E, F** for terminology and gene profile defining each topic in **Supplementary Table 3**). For visualization purposes, we assigned each spot to its dominant topic (defined as the topic with the highest estimated proportion per spot; **Figures 2E, F**). Spatial mapping of dominant topics per spot demonstrated relatively uniform composition across non-lesional samples (evidencing efficient batch-correction), in contrast to the marked heterogeneity observed across lesional samples, with each patient displaying distinct spatial patterns (**Figure 2F**). Aggregating the estimated topic proportions by immune versus stromal/epidermal topics revealed that non-lesional skin was predominantly stromal/epidermal, while lesional skin was largely immune (**Figure 2G**).

To characterize topic composition differences between paired non-lesional and lesional skin samples, we applied linear mixed effects modelling (41) on spot-level deconvolution data from all spots (n=5,562 spots, 5 patients, one biopsy per condition per patient), treating each spot as an independent transcriptomic unit defined by its cellular composition and spatial context, with patient-specific effects included as a random effect. This revealed differences in topic abundance per spot (p_adj < 0.05; log2FC: -4.06 to +6.62) with lesional and non-lesional tissues showing expected enrichment for immune and inflammatory topics and structural and epithelial topics, respectively (**Figure 2G**; **Supplementary Table 3**). These findings demonstrate that while the lesional tissue is highly heterogenous across different patients (**Supplementary Figure 1G**), each is characterized by increased immune cell infiltration when compared to the paired non-lesional tissue.

### Inter-patient variation in lesional architecture

**Figure 3:**
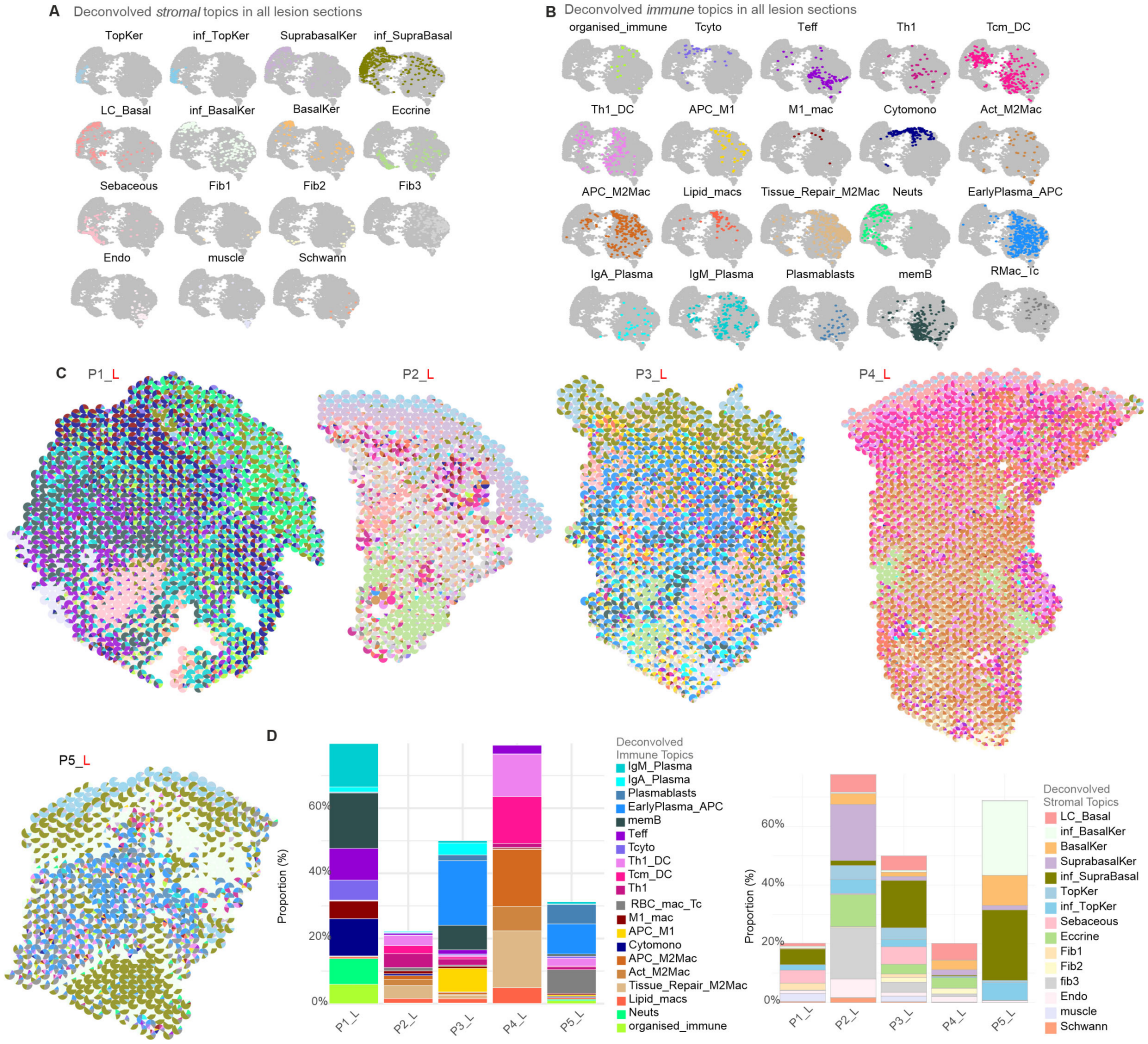
Cell type-associated topic deconvolution reveals patient-specific patterns of immune and stromal composition in lesional tissue. **(A, B)**, Separate UMAPs for each stromal **(C)** and immune **(D)** topic, with spots colored by topic dominance. **(C)** Spatial maps of each lesion with pie charts showing topic composition per spot. **(D)** Relative proportions of immune (left panel) and stromal (right panel) topics per lesion.

To dissect the heterogeneity of each lesion in greater detail, we subclustered only the lesional tissues (**Supplementary Figure 2A**) and used STdeconvolve (resolution K = 35) to obtain lesion specific topics. We identified 28 lineage-specific topics and six mixed topics with signatures from multiple cell types (**Figures 3A, B**; **Supplementary Figures 2B, C**; **Supplementary Table 3**).

**Figure 4:**
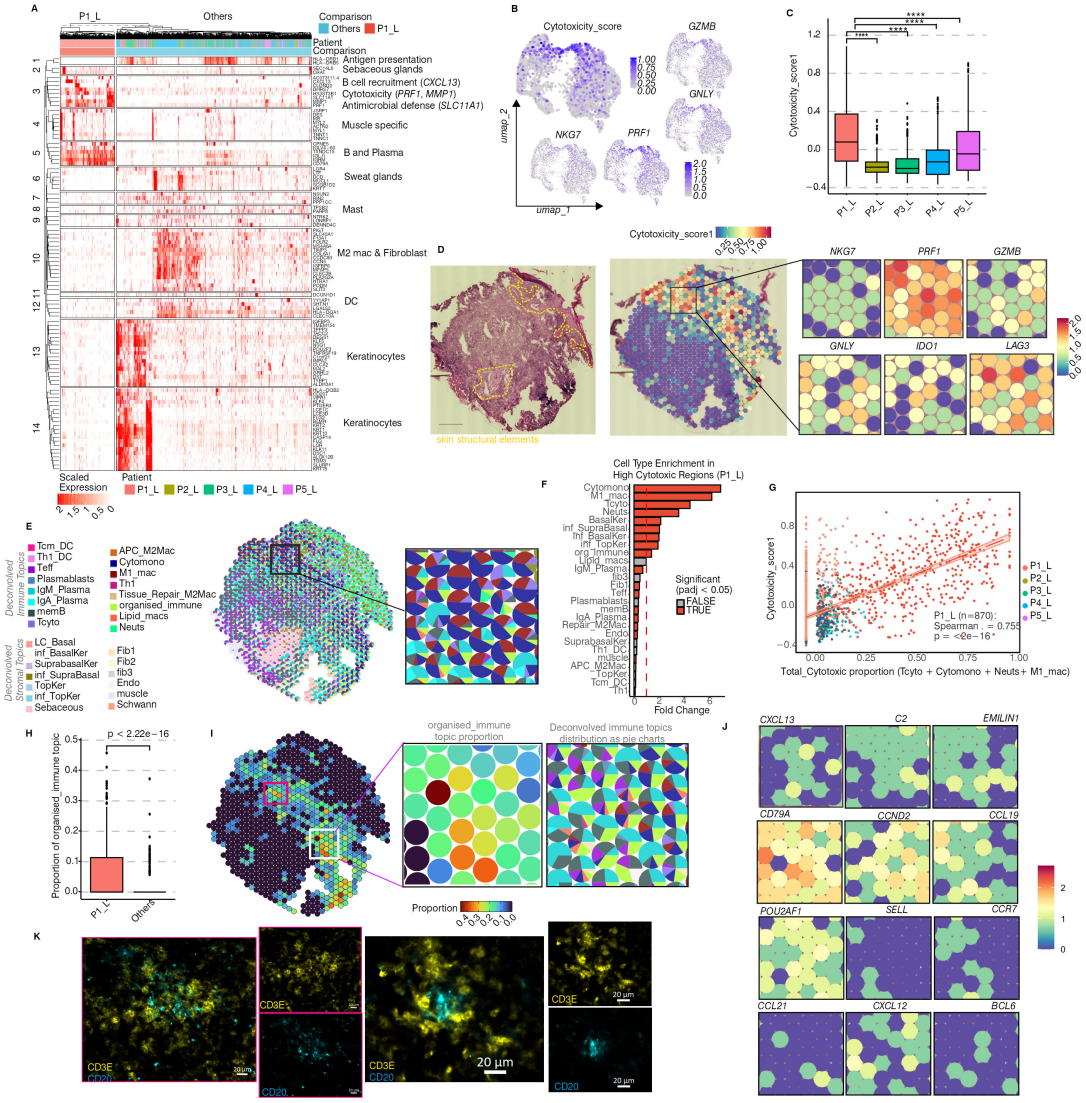
Cytotoxic immune response and tertiary lymphoid structures in P1. **(A)** Heatmap of top 100 DEGs in P1_L vs other lesional skin (P2-P5 L) (padj < 0.05, log2FC > ± 0.25; Wilcoxon) **(B)** UMAP of cytotoxicity signature in lesional skin (P1-P5). Top: composite score; right/below: four cytotoxic effector genes. **(C)** Cytotoxicity score across all patients (exploratory spot-level comparison, Wilcoxon; P1_L vs P2-P5 L). **(D)** Spatial map of cytotoxicity score in P1_L (middle), underlying H&E histology (left) and high-magnification spatial expression of selected genes from representative region(right). **(E)** Topic deconvolution (pie charts) of representative area (from D). **(F)** Fold enrichment of topics in high (≥Q3, top 25%) vs low (<Q3, bottom 75%) cytotoxic regions of P1_L (exploratory spot-level comparison, Wilcoxon with Bejamini Hochberg correction; red dotted line: log2FC =1). **(G)** Correlation between total cytotoxic cell abundance (Tcyto, Cytomono, M1_mac, Neuts) and cytotoxicity score. Points coloured by patient; P1_L (95% CI); ρ = Spearman’s rank. **(H)** Organised_immune topic proportion in P1 L vs P2-P5 L (Wilcoxon). **(I)** Organized_immune topic distribution in P1_L across full tissue (left), magnified region (middle; white square region enlarged), and other topics as pie charts (right). **(J)** Tertiary lymphoid structures (TLS) hallmark gene expression in representative areas from (**(I)**; pink and white square regions). **(K)** Multiplex immunofluorescence (IF)of two putative TLS from P1_L showing CD3E+ T cells (top) and CD20+ B cells (bottom) in magnified regions. Scale bar, 20µm.

**Figure 5:**
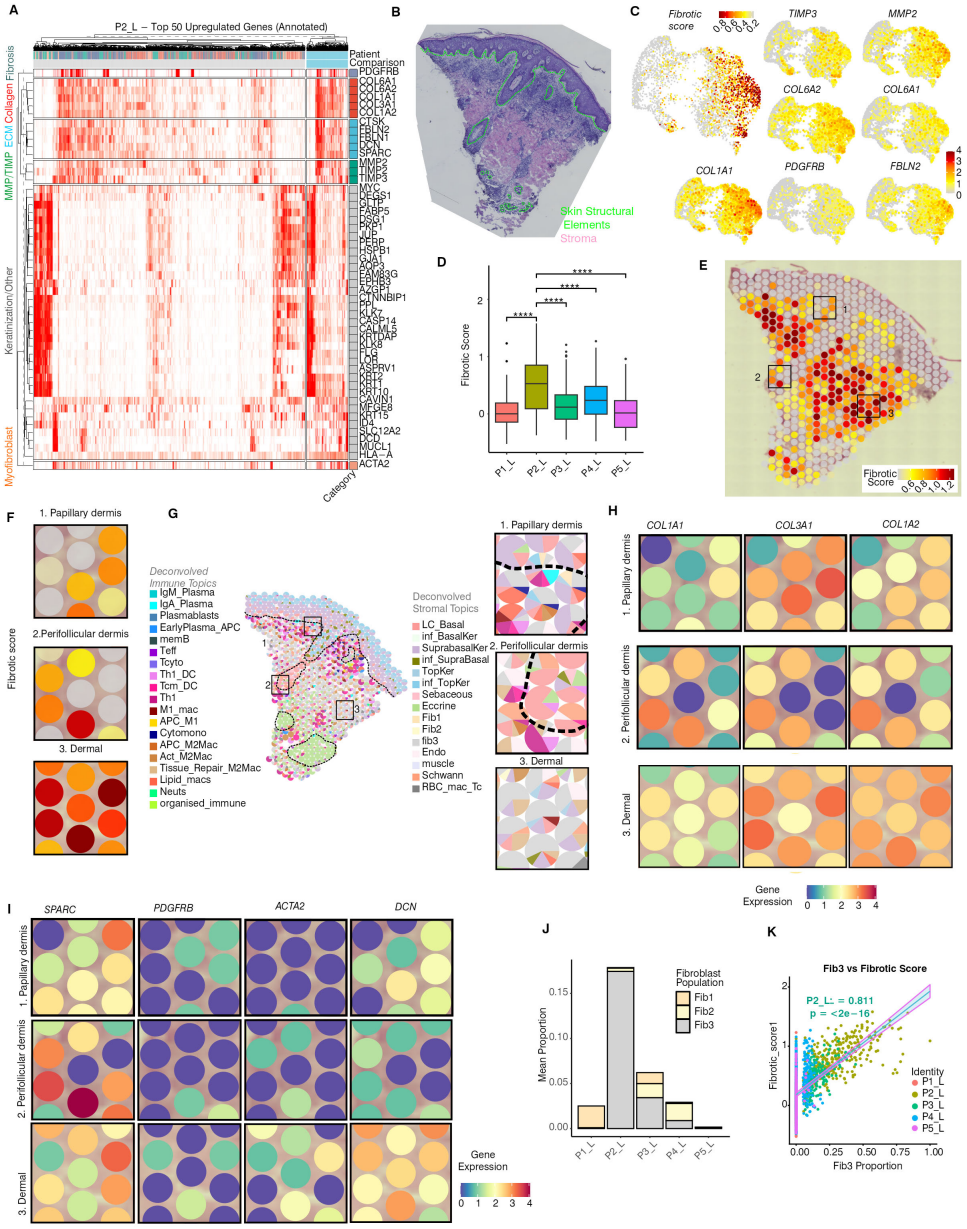
P2 shows extensive fibrotic remodeling with reduced immune infiltration. **(A)** Heatmap of top 50 upregulated genes between patient P2_L vs other lesional skin (P1, P3-P5 L) with functional annotation (padj < 0.05, log2FC > ± 0.25; Wilcoxon). **(B)** H&E histology from P2_L. green dotted line: skin structural elements: **(C)** UMAP of fibrosis signature (in lesional skin (P1-P5). Top: composite fibrosis score; right/below: 7 selected individual fibrotic genes. Colour scale: 25th-95th percentile). **(D)** Fibrotic score comparison across all lesional skin (exploratory spot-level comparison, Wilcoxon; P2L vs others). **(E)** Spatial map of fibrotic score in P2_L (colour scale: q25-q95 for P2 only) with three representative regions (black boxes): 1-papillary dermis, 2-perifollicular dermis, 3-dermal region. **(F)** Enlarged views of regions 1–3 showing compartment-specific patterns. **(G)** Cell-type-associated topic deconvolution (pie charts) on regions from **(F)**. **(H, I)** Spatial expression of selected fibrotic genes in regions from **(F)**. **(J)** Mean proportion of Fib1, Fib2 and Fib3 fibroblast deconvoluted topics in lesional skin (P1-P5). **(K)** Correlation between Fib3 proportion and fibrotic score across all spots (P1-P5). Points coloured by patient; trendline: P2_L (95% CI); ρ = Spearman’s rank).

**Figure 6:**
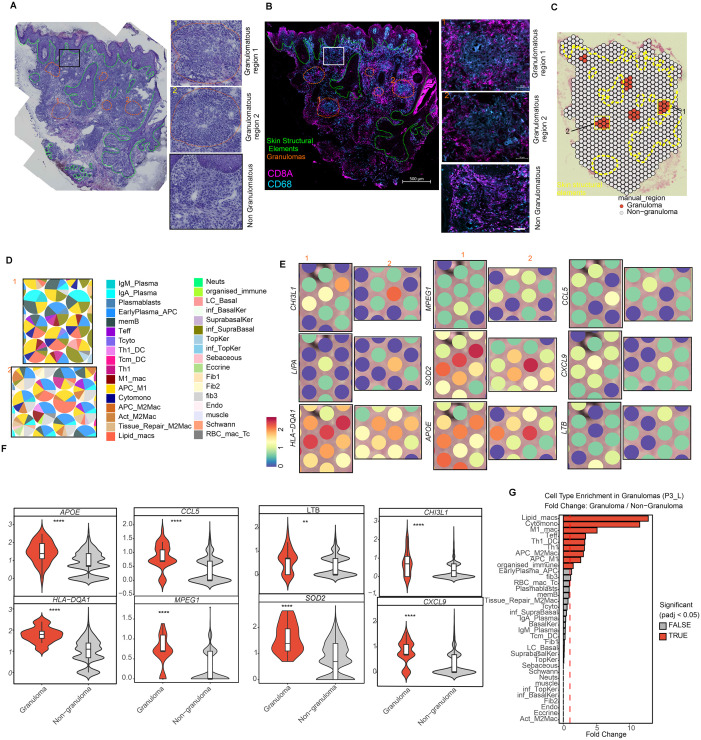
Patient P3 L exhibits distinct granulomatous inflammation with enriched antigen presentation machinery and lipid-associated macrophages. **(A)** H&E staining of P3_L tissue. Right: magnified views of representative granulomas (regions 1-2) and 1 non granulomatous region (black box; 3rd row). **(B)**, Multiplex IF staining of CD68 (myeloid; blue) and CD8 (T cells; magenta) of P3_L. Right: high-magnification views of regions 1–2 and non-granulomatous region from **(A)**. **(C)** Spatial map of P3_L showing granulomatous (red) and non-granulomatous (gray) regions based on underlying histology. Yellow dotted lines and black boxes: structural elements and regions 1-2 [from **(A)**]. **(D)** Cell-type-associated topic deconvolution (pie charts) for regions from **(C) (E)** Spatial expression of selected marker genes in regions 1 and 2 from **(C)**. **(F)** Expression of genes from **(E)** in granulomatous (red) vs non-granulomatous (gray) regions (exploratory spot-level comparison, Wilcoxon). **(G)** Fold change in topic proportions between granulomatous and non-granulomatous regions in P3_L. Red: significant enrichment (exploratory spot-level comparison, padj < 0.05, Wilcoxon); red dotted line drawn at log2FC =1.0. In panels **(C, D)** granulomatous regions marked with orange dotted lines; skin structural elements (epidermis, hair follicles) outlined in green. **p < 0.01, ****p < 0.0001.

**Figure 7:**
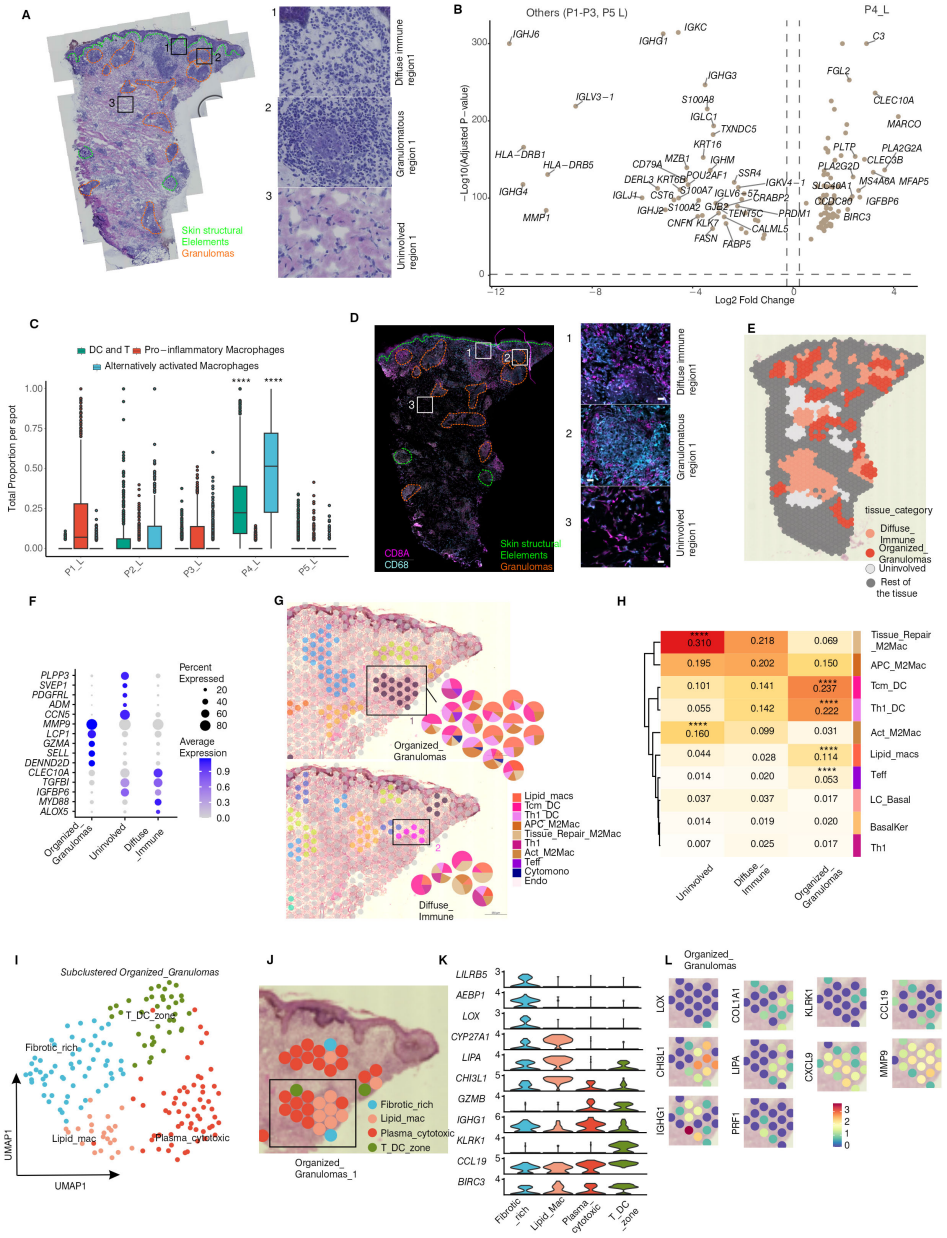
Patient P4_L demonstrates M2-polarized macrophage microenvironment with embedded granulomas. **(A)** H&E of P4_L with representative magnified regions 1–3 showing granulomas, diffuse immune infiltrate and uninvolved annotated regions. **(B)** DEGs between P4_L and other lesional samples (P1-3 & P5 L; top 50 genes labelled, Wilcoxon test). **(C)** Comparison of macrophage, T and DC topic proportions across patients (exploratory spot-level comparison, pairwise Wilcoxon test). **(D)** CD8A/CD68 multiplex IF on P4_L (right) with magnified regions 1–3 from **(A)** (left). **(E)** Spatial annotation map categorizing immune infiltration patterns (organized granuloma, diffuse immune, uninvolved, other). **(F)** Top 5 DEGs per annotation category. **(G)** Representative organized immune (area 1) and diffuse immune (area 2) regions with deconvolved cell-type-associated topic pie charts showing distinct compositions. **(H)** Mean cell-type-associated topic proportions across annotation categories (top 10 enriched types, pairwise exploratory spot-level comparison, Wilcoxon). I-J. UMAP subclustering of organized immune spots **(I)** and spatial projection onto area 1 **(J)**. **(K, L)**. Top marker genes for subclusters [stacked violin plots, **(K)**] and their spatial expression in organized granuloma area 1 **(L)**. (****p < 0.0001). In **(A, D)** Granuloma: orange dotted lines; skin structural elements (epidermis, hair follicles): green.

**Figure 8:**
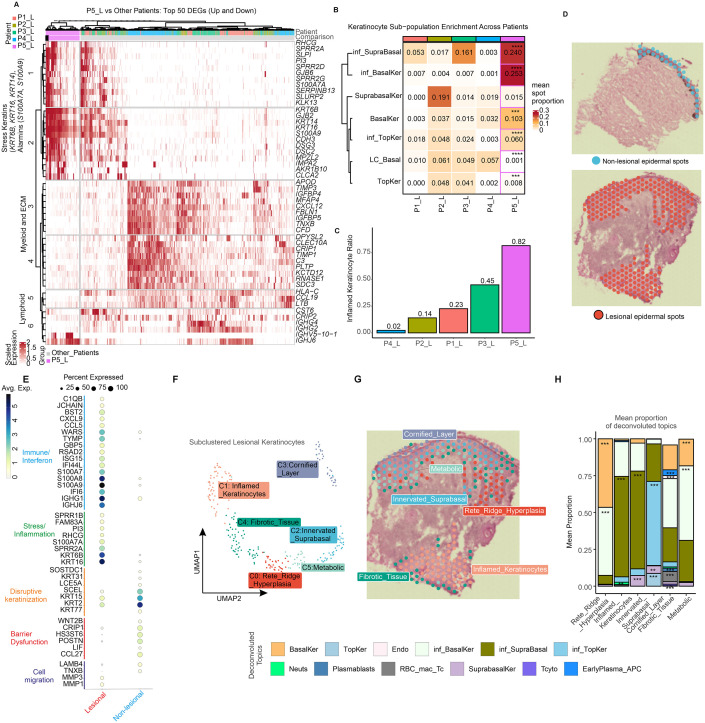
Spatial and molecular characterization of pseudoepitheliomatous hyperplasia in patient P5_L. **(A)** Heatmap showing top 50 DEGs in P5_L vs other lesional skin (P1- P4_L); annotated by functional category. **(B)** Heatmap of keratinocyte subpopulation enrichment across patients (mean proportions; exploratory spot-level comparison, pairwise Wilcoxon). **(C)** Mean proportion of inflamed keratinocyte subtypes (inf_BasalKer, inf_SupraBasal, inf_TopKer) relative to total keratinocytes (inflamed and non-inflamed) per patient. **(D)** Spatial map of P5 with H&E showing epidermal spots. Blue: P5_NL epidermis; red: P5_L epidermis. **(E)** Marker gene expression between P5_L and P5_NL epidermal spots from **(D)** grouped by biological function. **(F)** UMAP of P5_L epidermal spots showing clusters. **(G)** Spatial distribution of the six epidermal subclusters from **(F)** overlaid on H&E. **(H)** Mean proportions of deconvoluted topics (>1% abundance) per epidermal subcluster in **(F)**. Exploratory spot-level comparison, Wilcoxon. *p<0.05, **p<0.01, ***p<0.001.

Topic proportions per spot revealed distinct immunological features across patients (**Figures 3C, D**). P1 exhibited enrichment of topics associated with B/plasma cells, diverse T cell subsets, proinflammatory macrophages, and neutrophils, suggesting a proinflammatory response. P2 showed high stromal topic contribution with Th1 predominating among immune topics. P3 was characterized by antigen-presenting topics while P4 had predominantly topics associated with alternatively activated macrophage and T cell-DC mixed topics. P5 displayed the highest proportions of plasma cell, plasmablast, Cytomono, and inflammatory keratinocyte topics. We then used individual patients as exemplars of their dominant response, as the basis for further analysis (**Figures 4**–**8**), as appropriate for an exploratory case-based spatial analysis.

### Cytotoxic T cell responses and lymphoid neogenesis

Patient P1 was enriched for inflammatory cells populations in lesional tissue (**Supplementary 3A**) and differential expression analysis (Wilcoxon rank sum test; Bonferroni FDR correction) against all other patients revealed elevated expression of the cytotoxic effector (*PRF1*), B cell and plasma cell-associated genes (*IGLV4-60*, *TXNDC15*, *IGLJ1*, *IGHM*, *CD79A*), B cell-attracting chemokine *CXCL13*, enrichment of antimicrobial response genes (*HS3ST3B1*, *SLC11A1*), and ECM remodelling enzymes (*MMP1*) (**Figure 4A**).

We first calculated a cytotoxicity score using the average expression of established cytotoxic markers (*GZMB*, *GNLY*, *NKG7*, *PRF1*) to compare cytotoxic activity across all patient lesions (**Figure 4B**; **Supplementary Figure 3B**). P1 exhibited a higher cytotoxicity score compared to all other patients (**Figure 4C**). Spatial mapping revealed that high-cytotoxicity areas (defined as >75th percentile of cytotoxicity score) spanned the papillary dermis and extended into the reticular dermis. These regions showed significant enrichment of interferon-responsive genes (*ISG15*, *RSAD2*, *CXCL11*), antimicrobial factors (*CTSL*, *SLC11A1*), and immune check point markers (*IDO1*, *LAG3*) (**Figure 4D**; **Supplementary Figure 3C**). Deconvolution of P1-specific high cytotoxicity spots revealed enrichment (p < 0.001, Wilcoxon) of lineage-enriched topics: Cytomono (6.93-fold), Tcyto (4.48-fold), M1_mac (6.13-fold), and Neuts (3.53-fold) (**Figures 4E, F**; **Supplementary Figure 3D**; **Supplementary Table 5**). The combined proportions of these four topics correlated significantly with cytotoxicity scores in P1 (**Figure 4G**) but not in other patients, indicating that this specific cellular composition is uniquely associated with P1.

The second major feature associated with P1 was the presence of a mixed topic (organised_immune) with co-occurrence of genes associated with B cells, T cells and APC. This topic is indicative of tertiary lymphoid structures (TLS), ectopic lymphoid aggregates that form at sites of chronic inflammation. Among all patients, P1 exhibited the highest proportion of the organized_immune topic (p < 0.001, Wilcoxon) (**Figure 4H**), with spatial distribution concentrated in the reticular dermis (**Figure 4I**). To characterize these microenvironments further, we mapped canonical TLS markers, revealing coordinated expression of *CXCL13* and *CD79A* (B cell recruitment), *POU2AF1* (a transcriptional co-activator required for germinal centre formation), C2 (complement-mediated antigen presentation), *CCND2* (cell cycle activity), lymphocyte recruitment chemokines and receptors (*SELL*, *CCL19*, *CCR7*, *CXCL12*), extracellular matrix (Fibroblastic reticular cell marker, *CCL21* and *EMILIN1*) and *BCL6* (a marker of mature germinal centres) (**Figure 4J**). The coordinated spatial expression of these markers confirms the presence of organized TLS in P1. To confirm at the protein level, immunohistochemistry revealed aggregated CD20^+^ B cells forming a central follicular core surrounded by CD3^+^ T cells, indicative of organised TLS and recapitulating the characteristic zonation of organized lymphoid structures (42, 43) (**Figure 4K**). Notably, P1 had a higher parasite load than others (microscopy grade 4+, kDNA PCR ct= 9.94; **Table 1**; **Supplementary Figure 3E**), though we do not infer causation. In summary, P1 was characterized by high skin parasite load, a robust cytotoxic immune response, and the presence of TLS.

### Fibrotic remodelling with preserved stromal architecture

Comparison of P2 to all other lesional samples (DE**;** Wilcoxon rank sum test; Bonferroni FDR correction) revealed upregulation of collagen genes (*COL6A1*, *COL6A2*, *COL1A1*, *COL3A1*, *COL1A2*), fibrotic markers (*PDGFRB*), ECM components (*FBLN2*, *FBLN1*, *DCN*, *SPARC*), remodelling enzymes (*MMP2*, *TIMP2*, *TIMP3*), and the myofibroblast marker *ACTA2*, collectively indicating active wound healing and fibrotic remodelling (**Figure 5A**). These findings corroborate the clinical and histological presentation of this patient, including a prolonged lesion duration of 26 months with hyperpigmentation and evident fibrosis (**Figure 5B**).

To validate this observation, we created a fibrotic score based on average expression of *COL1A1, COL1A2, COL3A1, COL6A1, COL6A2, MMP2, TIMP2, TIMP3, ACTA2, PDGFRB, SPARC, FBLN1, FBLN2, DCN* genes and compared scores across different patients. P2 had the highest fibrotic score (**Figures 5C, D**; **Supplementary Figure 4**). Spatial plotting of the fibrotic score revealed extensive fibrosis in the reticular dermis consistent with stromal eosin staining in histology (**Figures 5B, E**). To look at spatial gene expression of fibrotic genes and whether they differed across different sites we then concentrated on three areas in the dermis, 1, papillary dermis, 2, perifollicular dermis and 3, reticular dermis (**Figure 5F**). The papillary and perifollicular dermis were also associated with immune infiltrate (**Figure 5G**) and therefore the reticular dermal spots which had the lowest immune composition had the highest fibrotic score. While most of the fibroblast genes were expressed in all three areas (**Figures 5H, I**), transcripts for the myofibroblast gene *ACTA2* and fibrosis gene *PDGFRB* were of lowest abundance in the papillary dermis and perifollicular dermis, respectively.

Among the three fibroblast-enriched topics (Fib1-3), P2 showed enrichment of Fib3, which expressed ECM remodelling genes (*ELN*, *PODN*, *MFAP4*, *THBS2*), matrix-degrading enzymes (*MMP2*, *CTSK*), and myofibroblast markers (*SFRP2*, *LRRC15*) (**Figure 5J**). The proportion of Fib3 strongly correlated with fibrotic score (Spearman’s ρ = 0.8, p < 0.001), suggesting this population is associated with fibrotic remodelling (**Figure 5K**). Together, these findings demonstrate that P2 is characterized by extensive fibrotic remodelling and reduced immune infiltration, as might characterize a wound healing phenotype.

### Granulomatous inflammation with proinflammatory characteristics

Histological examination of P3 revealed extensive immune infiltration with multiple discrete granulomas throughout the dermis. Granulomas were characterised by an epithelioid CD68^+^ macrophage-rich core and a lymphocytic cuff, with abundant CD8A^+^ cells (**Figures 6A, B**). To dissect the molecular and cellular features of these granulomas further, we manually annotated each granuloma in the spatial transcriptomics data using the underlying tissue morphology (**Figure 6C**). Spatial maps of the proportion of deconvoluted topics in spots representing two representative granulomas revealed enrichment of macrophage, B cell and T cell topics, with spatial heterogeneity in distribution within each granuloma (**Figure 6D**). Spatial gene expression mapping showed coordinated expression of markers for foamy macrophages (*CHI3L1*, *LIPA*, *APOE*), MHC class II molecule (*HLA-DQA1)*, antimicrobial genes (*MPEG1*, *SOD2*), and chemokines involved in T cell recruitment and activation (*CCL5*, *CXCL9*, *LTB*) (**Figure 6E**). These genes were significantly enriched in granulomatous areas compared to non-granulomatous regions (**Figure 6F**). Topic enrichment analysis revealed that granulomas were predominantly composed of lipid-associated/tissue adapted macrophages (*CHIT1*, *CHI3L1*, *LIPA*, *CYP27A1*, *CD68*; 12.8-fold, p < 0.001), cytotoxic monocytes (*APOE*, *PRF1*, *LYZ*; 11.5-fold, p < 0.001), proinflammatory M1 macrophages (*TCN2*, *G0S2*, *CD68*; 5.0-fold, p < 0.001), and effector T cells (*FCMR*, *CXCR3*, *LTB*, *CCL5*; 3.0-fold, p < 0.001) (**Figure 6G**).

Additionally, differential gene expression analysis between P3 (Wilcoxon rank sum test; Bonferroni FDR correction) and all other patients identified a marked B cell infiltration in this patient with abundant transcripts for class-switched immunoglobulin genes (**Supplementary Figures 5A–C**). Together, these findings demonstrate that P3 is characterized by the presence of organized granulomas with proinflammatory/remodelling characteristics, and that are embedded in a microenvironment with prominent B cell infiltration.

### Granulomatous inflammation with M2 myeloid cell polarization

Similar to P3, histology from P4 indicated a prominent granulomatous response (**Figure 7A**), but differential gene expression analysis (Wilcoxon rank sum test; Bonferroni FDR correction) revealed a strikingly different surrounding microenvironment (**Figure 7B**; **Supplementary Figure 6A**). All P4 spots had enrichment of lysosomal genes (*MARCO*, *CTSH*, *MPEG1*, *TMEM176B*, *CTSZ*), MHC-II and antigen-presenting machinery genes (*CD4*, *HLA-DMB*, *HLA-DPA1*, *IRF8)* chemokines and immune mediators (*CXCL9*, *TRAC*, *FYB1*, *C1QA*, *LCP1*, *FCER1G*, *CXCL14*), complement genes (*SERPINB1*, *PSGL2*, *C3*), myeloid markers (*CLEC10A*, *MARCO*, *MS4A6A*), lipid metabolism genes (*PLA2G2D*, *PLA2G2A*, *PLB7*, *SLC40A1*), and ECM remodelling factors (*TGFB1*, *TIMP1*, *TIMP3*, *FN1*, *CLEC3B*, *MFAP5*, *IGFBP6*), collectively indicating dominant infiltration by tissue-resident macrophage phenotypes with active tissue repair and remodelling programs. Conversely, P4 showed reduced expression of B cell and plasma cell markers (*IGHG1-4*, *POU2AF1*, *CD79A*, *DERL3*, *PRDM1*) and keratinocyte inflammatory genes (*KRT6B*, *S100A2/7/8*), suggesting minimal plasmacytic and epithelial inflammation compared to other patients (**Figure 7B**). These gene expression patterns suggested enrichment of alternatively activated myeloid phenotypes in P4.

To quantify macrophage phenotypes and T cell-dendritic cell interactions, we compared the combined proportions of macrophage and T-DC mixed topics across patients. P4 exhibited higher proportions of topics enriched for alternatively activated macrophages (APC_M2Mac, Act_M2Mac, Tissue_repair_M2Mac, and Lipid_macs per spot) and the mixed Th1_DC and Tcm_DC topics compared to all other lesions (Wilcoxon rank-sum test, p < 0.001) (**Figure 7C**). In contrast, total proinflammatory macrophage topics (Cytomono, M1_mac, and APC_M1) were not enriched in P4.

To determine whether this tissue microenvironment influenced granuloma form or function, we first identified granulomas in P4 by immunostaining, and annotated four distinct tissue compartments based on morphology (1): organized granulomas with defined epitheliod macrophages and lymphocytic cuff (Organized_Granulomas, **Figure 7D-E**) (2), diffuse peri-granuloma infiltrate without clear organization (Diffuse_Immune) (3), uninvolved stroma with minimal immune infiltration (Uninvolved) (4), remaining tissue (**Figure 7E**). These regions were associated with distinct molecular signatures reflecting lymphocyte recruitment, cytotoxicity and tissue remodelling in organized granulomas (*MMP9*, *LCP1*, *GZMA*, *SELL, DENND2D*) myeloid cell surveillance and regulation in diffuse immune areas (*CLEC10A*, *TGFB1*, *IGFBP6*, *MYD88*, *ALOX5*), and ECM genes in uninvolved stromal regions (*CCN5*, *ADM*, *PDGFRL*, *SVEP1*, *PLPP3*) (**Figure 7F**).

Mapping deconvoluted topics onto a representative granuloma and its surrounding diffuse immune region revealed higher proportions of lipid-associated macrophages, cytotoxic monocytes, effector T cells, and Th1-DC interactions within the organized granuloma compared to adjacent diffuse infiltrate (**Figure 7G**). To systematically characterize cellular enrichment patterns across all annotated regions, we performed topic enrichment analysis comparing each region to organized granulomas. Tissue repair M2 macrophages (*MARCO*, *MS4A6A*, *CLEC10A*, *CD68*; 4.5-fold; padj<0.0001) and activated M2 macrophages (*CCL13*, *VSIG4*, *CD14*; 5.16-fold; padj<0.0001) were enriched in uninvolved stroma, while antigen-presenting M2 macrophages (*PLA2G2D*, HLA*-DQA1, CD68*) predominated in diffuse immune areas (1.34-fold; padj<0.001). In contrast, Tcm-DC (*CCL19*, *IL7R*, *TRBC2*, *CLEC10A; ~*1.5 fold, padj<0.0001), Th1-DC (*CXCL9*, *IL4I1*, *CD4*, *CD3D; ~*1.5 fold, padj<0.0001), and lipid-associated macrophages (4.12-fold, padj<0.0001) were most abundant in organized granulomas when compared to the neighbouring diffuse immune infiltrate (**Figure 7H**; **Supplementary Figure 6B**).

Finally, to identify potential spatial zonation within granulomas, we subclustered all granulomatous spots and examined their spatial distribution (**Figures 7I, J**). Based on gene expression profiles, we identified four distinct zones (**Figure 7I**; **Supplementary Figure 6C**) (1): a fibrotic zone characterized by ECM genes (*FN1*, *COL3A1*, *COL1A2*, *COL1A1*, *TIMP1*, *MMP2*) (2); a plasma cell-rich cytotoxic zone expressing immunoglobulins and cytotoxic effectors (*IGHG1*, *IGHA1*, *JCHAIN*, *GZMB*, *PRF1*) (3); a T cell-dendritic cell interaction zone marked by chemokines and T cell markers (*CCL19*, *CXCL9*, *GBP5*, *CCL17*, *CD3D*, *CD3E*); and (4) a lipid-associated macrophage zone with markers of foamy macrophages and tissue remodelling (*CHI3L1*, *LIPA*, *PLIN2*, *MMP9*, *WARS*, *APOE*, *CD68*). Spatial mapping of these subclusters onto a representative granuloma (organized_granuloma_1) revealed a structured architecture with distinct spatial compartmentalization (**Figure 7J**) and gene expression patterns (**Figures 7K, L**). The fibrotic zone showed highest expression of *LILRB5*, *AEBP1*, *COL1A1* and *LOX*. The lipid macrophage zone expressed *CYP27A1*, *LIPA*, and *CHI3L1*. The plasma-cytotoxic zone exhibited elevated *GZMB* and *IGHG1* and the T-DC zone showed enrichment of *KLRK1* and *CCL19*. Notably, *BIRC3*, an anti-apoptotic factor involved in NF-κB signalling, showed ubiquitous expression across all granuloma subclusters, indicating active cell survival programs throughout the organized structure. Spatial gene expression mapping confirmed these distinct molecular territories. Notably, we observed a broader *CXCL9* and *MMP9* expression indicating sustained immune activation and tissue remodelling throughout the granulomatous region (**Figure 7L**).

Together, these findings demonstrate that P4 exhibits a unique immunological architecture characterized by organized granulomas embedded within an M2-polarized tissue microenvironment. While P4 granulomas share structural features with P3, including epithelioid macrophages and lipid-associated macrophage signatures, differential expression analysis between P3 and P4 granulomas (**Supplementary Figure 6D**) revealed that P3 granulomas had more abundant transcripts for MHC class II genes (*HLA-DRB1*, *HLA-DRB5*) and immunoglobulins (*IGHG1*, *IGHG3*, *IGHG4*, *IGHJ6*, *IGHJ2*). P4 granulomas, in contrast, show higher complement activation (*C3*) and chemokine-mediated T cell recruitment (*CXCL9*, *CCL19*). Thus, P3 and P4 represent distinct forms or stages of granulomatous inflammation distinguishable by gene expression.

### Pseudo-epitheliomatous hyperplasia with marked immune infiltration and interferon response

The preceding analyses revealed distinct immune-centric pathologies across the four (P1-P4) patients. We next examined P5, the only patient with confirmed *L. tropica* infection. Comparative transcriptomic analysis of P5 against all other patient lesions (DE**;** Wilcoxon rank sum test; Bonferroni FDR correction) revealed upregulated genes associated with keratinocyte hyperproliferation (*KRT6B*, *KRT14*, *KRT16*), antimicrobial defense and alarmins (*S100A7A/9, SLPI*, *PI3*, *SERPINB13*), cornification and cell adhesion and junction proteins (*DSG3*, *DSC2*, *CDH3*, *GJB2/6*) (**Figure 8A**) indicating epidermal hyperplasia as the dominant pathological feature of P5. Conversely, P5 showed reduced expression of ECM genes (*TIMP3*, *IGFBP4*/5 *MFAP4*, *FBLN1*, *TIMP1*), tissue-resident myeloid markers (*CLEC10A*, *C3*, *RNASE1*), immune genes (*HLA-C*, *CCL19*, *LTB*), and class-switched immunoglobulins (*IGHG2*, *IGHG4*).

Inflamed keratinocyte topics were strikingly enriched across all three epidermal layers in P5 (inf_BasalKer, BasalKer, inf_SupraBasal, inf_TopKer; padj<0.05, Wilcoxon; **Supplementary Table 6**; **Figure 8B**). This pan-epidermal inflammatory signature indicates inflammatory hyperplasia rather than proliferative expansion Quantitatively, P5 exhibited the highest inflamed-to-total keratinocyte ratio among all patients (0.82; **Figure 8C**).

To characterize inflamed keratinocyte signatures, we manually annotated epidermal regions based on tissue morphology and extracted gene expression profiles from matched lesional (P5_L) and non-lesional (P5_NL) epidermal spots (**Figure 8D**). Differential expression analysis between P5_L and P5_NL epidermis revealed disruption of five major biological programs (**Figure 8E**; **Supplementary Table 7**): (a) immune/interferon infiltration, with increased expression of interferon-stimulated genes, immunoglobulins, immune mediators, and DAMPs; (b) stress and inflammation, with elevated stress keratins, cornified envelope precursors, and antimicrobial peptides; (c) disrupted differentiation, with reduced terminal differentiation markers; (d) barrier dysfunction, with loss of homeostatic signaling molecules including CCL27 and LIF, and structural genes; and (e) altered cell migration with upregulation of matrix remodelling genes. To identify functionally disrupted keratinocyte compartments, we subclustered all epidermal spots from P5_L revealing six spatially distinct microenvironments (**Figures 8F, G**): with cluster-specific signatures (**Supplementary Figure 7A**; **Supplementary Table 8**).

Finally, we examined the deconvolved topic composition for each of the six keratinocyte microenvironments (**Figure 8H**; **Supplementary Table 9**, Wilcoxon, padj<0.05) and confirmed by spatial mapping that each reflected distinct neighbourhood (**Supplementary Figure 7B**). Together, this analysis demonstrates that the epidermis of P5, a 1-month-old *L. tropica* infection, exhibits complex inflammatory hyperplasia characterized by six spatially distinct epidermal microenvironments, each with unique molecular signatures and predicted cellular compositions. This represents one of several active tissue programmes identified in this patient and may reflect either parasite-specific and/or patient-specific pathology.

## Discussion

**Figure 9:**
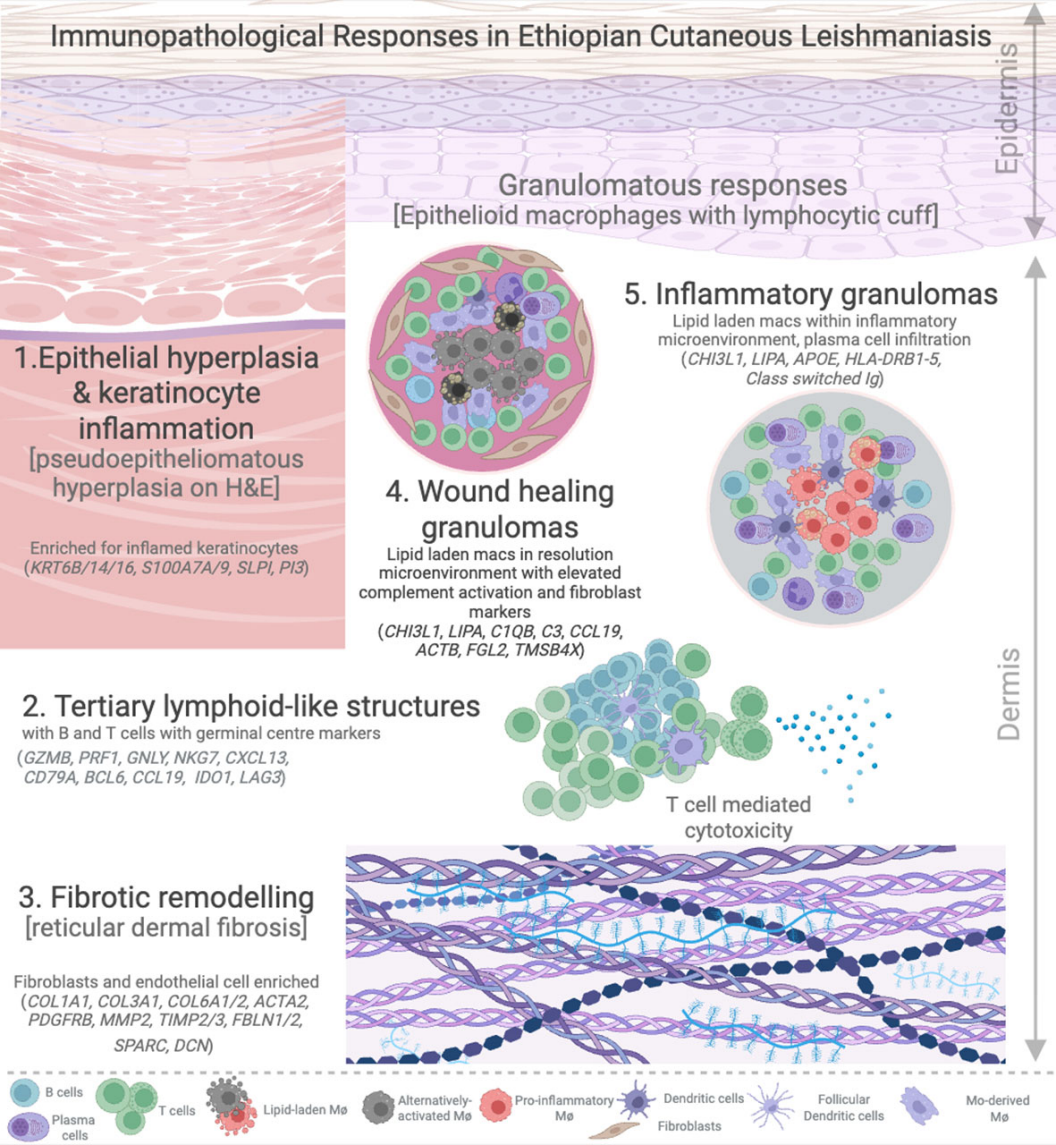
Five different candidate immune responses in Ethiopian CL.

In this study, we applied spatial transcriptomics to paired lesional and non-lesional skin biopsies from five Ethiopian patients with CL to define the cellular and molecular landscape of lesional tissue. While spatial transcriptomics has been applied to experimental human *L. major* infection (36) and *L. donovani*-associated CL in Sri Lanka (37, 38), these studies did not focus on the identification of distinct immunological niches associated with chronic lesions. In the current study, we describe five candidate tissue-level immunopathological programs across Ethiopian CL lesions (summarised in **Figure 9**).

A key feature of our study design was the use of paired lesional and non-lesional biopsies from the same individual, which allowed us to distinguish disease-associated transcriptional programmes from baseline skin signatures while controlling for inter-individual variation. This approach revealed enrichment of immune and inflammatory programmes in lesional tissue across all five patients, despite their markedly different immunopathological profiles. While this comparison allowed us to interpret patient specific immune signatures selective for the lesional site, our non-lesional biopsies may not represent completely healthy skin.

Several candidate immune programmes showed associations with lesion characteristics: P2’s fibrotic profile coincided with the longest lesion duration (26 months); P3 and P4 both showed granulomatous features despite distinct surrounding microenvironments; and P5, the only confirmed *L. tropica* case, showed prominent epithelial hyperplasia. Additional patterns included neutrophil enrichment in P1 and P5 consistent with super-infection, and sebaceous gland/IgA plasma cell enrichment in mucosal lesions (P1, P3). Given the small cohort size and incomplete species identification, these associations remain hypothesis-generating and should not be interpreted as causal or species-specific.

The presence of TLS in P1 represents, to our knowledge, the first report of lymphoid neogenesis in a CL lesion. Immunohistochemistry confirmed characteristic TLS architecture comprising B cells, T cells and a coordinated spatial expression of *CXCL13*, germinal centre and fibroblastic reticular cell markers. TLS formation in chronic infections often correlates with sustained antigen presence and attempts at local immune control (44). Paradoxically, P1 exhibited the highest parasite burden among all patients despite high cytotoxicity scores and elevated interferon-stimulated genes in TLS-adjacent regions. The co-occurrence of high parasite burden and TLS in P1 raises the possibility of a relationship between parasite persistence and lymphoid neogenesis, though whether this is causal or reflects lesion duration, severity, or anatomical location remains to be tested in larger cohorts. Our data suggest the need for further exploration of the role of TLS in CL pathogenesis, to determine whether they contribute to host protection or are associated with exaggerated immunopathology and chronicity.

Organized granulomas were prominent in P3 and P4, characterized by epithelioid macrophage cores enriched for lipid-associated macrophages and surrounded by lymphocytes. These macrophages expressed *CHIT1* and *CHI3L1*, consistent with lesional macrophages observed in *L. donovani* CL (37) and foamy macrophage phenotypes described in multiple sclerosis (45) and coronary heart disease (46). The accumulation of lipid-associated macrophages is a hallmark of chronic infectious granulomatous diseases, reflecting sustained phagocytic activity and lipid metabolism dysregulation (47, 48). In tuberculosis, similar lipid-associated macrophage populations reside on granuloma boundaries (49) and may serve as niches for pathogen persistence (50).

Our spatial analysis revealed internal zonation in P4 granulomas with functional specialization including fibrotic zones, lipid-macrophage zones, T cell-DC interaction zones, and plasma-cytotoxic zones, consistent with spatial studies in microbial infection (49), Strikingly, the surrounding tissue microenvironments of P3 and P4 were molecularly distinct: P3 granulomas were embedded in a proinflammatory milieu characterized by high MHC-II expression, class-switched immunoglobulins, and plasma cell infiltration, while P4 granulomas were embedded within an M2-polarized wound healing microenvironment, with elevated complement activation, distinct chemokine expression patterns and fibroblast markers, illustrating that granulomatous microenvironments across Ethiopian CL can be molecularly heterogeneous. The organised zonation observed in P4, with *CXCL9* and *CCL19* upregulation, is reminiscent of the later stages of granuloma evolution observed in cutaneous sarcoidosis (51) and healing phase granuloma in *L. braziliensis*-infected macaque models, where fibroblasts proliferate at the granuloma periphery and invade granuloma structures (52).

Our observation of granuloma heterogeneity parallels findings in infections by other intracellular pathogens such as *Mycobacterium tuberculosis*, where pulmonary granulomas enriched for B cells and fibroblasts differ substantially from visceral peritoneal omental granulomas dominated by myeloid cells (53), and in lung granulomas in macaques where different cellular compositions reflect high burden early granulomas enriched with plasma cells, mast cells versus low burden late granulomas enriched with cytotoxic T cells (48). The presence of organized and unorganised granulomas has been associated with Ethiopian *CL* lesions (28, 54) but our analysis is the first to describe their molecular heterogeneity.

In P5, epithelial hyperplasia emerged as the most prominent and enriched pathological feature when compared to other patients. Inflamed keratinocyte signatures comprised 82% of total keratinocyte abundance across all epidermal layers, with widespread expression of interferon-stimulated genes, stress keratins, and antimicrobial alarmins. Six spatially distinct keratinocyte microenvironments were identified. Enrichment of inflamed keratinocytes with neutrophil recruitment is a well-characterized feature of leishmaniasis pathogenesis (55). Importantly, while epithelial inflammation was the dominant feature of P5, substantial dermal immune infiltration was also present, consistent with previous histologic report of *L. tropica* infection, illustrating that multiple active tissue programs likely co-exist within individual lesions. Notably, P5 is the only confirmed *L. tropica* case in this cross-sectional cohort but whether the prominent epithelial response in this patient reflects *L. tropica*-specific biology (55), early disease kinetics, and/or host specific factors remains to be determined.

Our immunopathological findings are consistent with recent histologic descriptions of *L.aethiopica* infection (28) from 26 patients showing epidermal changes, organized granulomas and patchy inflammatory infiltrates. Critically, our spatial approach dissects the molecular architecture underlying these histopathological patterns, revealing the distinct tissue programs and cellular associations that might define the tissue level heterogeneity across Ethiopian CL.

Signs of bacterial superinfection were noted in P1 and P5, both of which showed neutrophil enrichment and cytotoxicity. Bacterial superinfection may therefore represent a potential confounder for the interpretation of these findings, as neutrophil-associated inflammation, epithelial hyperplasia, and antimicrobial responses may be driven by secondary bacterial infection rather than, or in addition to, the underlying *Leishmania* infection. This study design cannot distinguish between these possibilities.

This exploratory study also has the following limitations with respect to generalizability across patients with Ethiopian CL. The small sample size was small (n=5). Single biopsies from index lesions may not capture the entire lesional landscape. Incomplete *Leishmania* species identification and marked variation in parasite density in lesions limits inference about parasite-driven effects and/or an association between observed immune response and protection. Furthermore, all patients were young men of similar demographic background, limiting generalizability across sex and age. The patient-level analysis also prevents adequate separation of the effects of parasite species, lesion duration, anatomical site, parasite burden, bacterial superinfection, and host-specific factors. Whilst some observations allude to determinants of heterogeneity (e.g., P1 and P5 showed neutrophil enrichment consistent with super-infection; mucosal lesions (P1, P3) exhibited sebaceous gland and IgA plasma cell enrichment; and late-stage lesions P2 (26 months) and P4 (11 months) displayed higher proportion of tissue repair macrophages and fibroblast topics), these need to be treated with caution. Additionally, Visium spatial resolution does not achieve single-cell resolution, and reference-free deconvolution lacks single-cell validation. Future studies with larger, longitudinal cohorts should integrate high resolution spatial parasite and host immune mapping to determine how parasite variation and immune programs drive disease progression, treatment failure, and clinical heterogeneity.

In conclusion, our study provides new insights into the complexity of the immunopathological response in patients with CL in Ethiopia. By spatially dissecting granulomatous inflammation, cellular compositions, and stromal and epithelial remodelling programs, we establish a molecular framework for understanding CL pathogenesis that bridges descriptive histopathology with molecular immunology. These findings are hypothesis-generating and future studies with larger, longitudinal cohorts will be needed to determine whether the distinct spatial programs identified here represent stable pathotypes, reflect lesion stage, or are shaped by parasite species, host factors, or a combination of these. These findings may ultimately inform the use of tissue biomarkers for potentially guiding patient stratification and personalised immunomodulatory strategies tailored to distinct tissue programs in CL.

## Methods

### Ethics

The study protocol was approved by the Ethiopian National Research Ethics Review Committee (NRERC; 02/246/574/22), the University of Gondar Institutional Review Board (VP/RTT/05/156/2021), the Institute of Tropical Medicine Antwerp Institutional Review Board (1451/20), the Antwerp University Hospital Ethics Committee (UZA 624), and the Ethical Review Board of the Hull York Medical School (21-22.24). The study followed the Declaration of Helsinki, Good Clinical Practices and local regulations. Written informed consent was obtained from all participants (or from the parent/guardian for adolescents). The study is registered at ClinicalTrials.gov (NCT05332093; registered 8 March 2022).

### Patient recruitment and sample collection

We included the first seven participants enrolled in our Spatial CL study investigating host-parasite interactions in Ethiopian CL at the Leishmaniasis Research and Treatment Centre (LRTC) for 10x Visium analysis. Patients were enrolled at the University Hospital of Gondar (Gondar, Amhara Province, Ethiopia) between March-May 2022 with follow-up until November 2022. Detailed inclusion and exclusion criteria are available at Clinicaltrials.gov but briefly it included no previous CL history and no severe comorbidities like diabetes and autoimmune diseases.

*Leishmania* infection was verified by microscopy of Giemsa-stained skin slits and kDNA PCR of skin slit smears. Lesion parasite load was graded according to WHO guidelines on a 0–6 scale (56). Parasite genotyping by ITS-1 high-resolution melt PCR (9) and SureSelect (57) identified two *L. aethiopica* (P1, P2) and one *L. tropica* (P5) infection. Following clinical reassessment, one patient did not have a lesion biopsy representative of the complete lesion (i.e., ‘MCL’ diagnosis with potential nasal mucosal infection but lesion biopsies were taken from the non-mucosal areas near the cheek and nose bridge), and was therefore omitted. Another patient was later diagnosed with leprosy and therefore omitted. The remaining five patients (P1-P5) included two with mucosal involvement (P1, P3) and three with purely cutaneous lesions on the arm (P2), neck (P4), and leg (P5). Clinical data was collected and recorded in a REDCap database. After enrolment, 4mm and 2mm punch biopsies (KAI Medical, Solingen, Germany) were collected from lesional (index lesion) and non-lesional skin (upper thigh or shoulder), respectively. Biopsy sizes were selected to balance adequate tissue yield for spatial transcriptomics and histological assessment from the lesional site, while minimising patient discomfort at the non-lesional control site. Both samples were snap-frozen in liquid nitrogen within 15 minutes, and stored frozen until Visium processing (10X Genomics, CA, USA).

### Tissue processing and assessment of RNA quality

Frozen tissue blocks were sectioned at 10 μm thickness and initial sections from each sample were assessed for RNA quality (RNA Integrity Number, RIN; 2.2-8.7). Due to low RIN values, we adapted the 10x Genomics Visium FFPE protocol for frozen sections described by Mirzazadeh et al. (58), which has been validated for spatially resolved transcriptomic profiling of degraded and challenging fresh frozen samples. Post spatial profiling, no correlation was observed between RIN and DV200 values and median genes detected per spot suggesting that data quality was not compromised by RNA degradation, supporting the adaptation of FFPE protocol used for frozen sections.

Sections were fixed with 4% methanol-free formaldehyde (Thermofisher, Catalog number 28906) for 10 min at room temperature, washed, Hematoxylin and Eosin (H&E) stained and imaged as per Visium guidelines. Post imaging sections were de-crosslinked with a single wash of 100 μl 0.1 N HCl for 1 min. Subsequent steps followed standard Visium FFPE protocols to ensure probe-based transcript capture.

### Spatial transcriptomics

Following decrosslinking, hybridization, extension, and ligation steps, cDNA libraries were constructed using the Visium Spatial Gene Expression reagent kit (10x Genomics) adapted for partially fixed frozen sections. Sequencing was performed on an Illumina NovaSeq 6000 instrument. Raw FASTQ files were processed using SpaceRanger (10x Genomics) to align reads to GRCh38 and generate feature-barcode matrices. For the combined dataset, quality control was performed using the cleanCounts function (STdeconvolve v1.3.2), removing spots with fewer than 80 total counts and genes detected in fewer than 8 reads across all spots. This resulted in the removal of 16 low-quality spots (0.3% of total), retaining 5,562 spots across all ten sections (P1_H: 242, P1_L: 870, P2_H: 213, P2_L: 529, P3_H: 400, P3_L: 839, P4_H: 360, P4_L: 1,198, P5_H: 368, P5_L: 543). For the lesion-only analysis STdeconvolve default thresholds were applied (minimum 100 total counts, minimum 10 reads per gene), removing a further 14 spots (0.4% of lesional spots) and retaining 3,976 lesional spots for downstream deconvolution (P1_L: 870, P2_L: 526, P3_L: 839, P4_L: 1,198, P5_L: 543).

### Data integration, normalization, and clustering

Filtered gene-barcode matrices were loaded into Seurat and normalized using SCTransform (default parameters). Data were integrated across samples using integration anchors. Principal component analysis (PCA) was performed on the integrated data, and clusters were identified using the Louvain algorithm at a suitable resolution. UMAP was used for two-dimensional visualization of spatial domains.

### Deconvolution of Visium spots and visualization

Cell type abundance in each Visium spot was estimated using STdeconvolve (v1.3.2 (40)) a reference-free deconvolution tool using latent Dirichlet allocation to identify recurrent gene expression patterns (“topics”) representing putative cell-type-associated topics or states. The output provides gene expression profiles per topic and topic proportions per spot. We excluded highly abundant keratin and immunoglobulin genes to prevent dominant epithelial and plasma-cell transcripts from overwhelming topic inference; sensitivity analyses confirmed that major topic structures were preserved when these genes were retained/excluded. However, *IGHM* and *IGHA1* were reintroduced to discriminate non-class-switched (IgM+) and class-switched (IgA+) plasma cells/B cells.

Overdispersed genes were identified using the restrictCorpus() function (removeAbove = 0.95, removeBelow = 0.01, alpha = 0.05) to capture genes with high biological variability. To ensure adequate T cell subset representation, we supplemented the over dispersed (OD) gene set with curated T cell markers. The final gene set combined all OD genes with immune markers present in the dataset. Deconvolution was performed with k=26 topics for the combined non-lesion and lesion dataset and k=50 topics for the lesion-only dataset. Topic annotations were assigned based on manual inspection of the top marker genes for each topic, cross-referenced against known cell-type and cell-state gene signatures from the literature. K numbers were ascertained based on model perplexity and number of topics with mean proportion <5%. Topics with highly similar marker gene profiles were identified by manual inspection of top marker genes and merged by summing spot-level proportions and averaging gene expression profiles. Specifically, duplicate topics merged in the combined dataset included two TopKer topics and three InflammKer topics, resulting in a final set of 23 topics. In the lesion-only dataset, merged topics included: two inf_TopKer, two IgM_Plasma, two inf_BasalKer, two EarlyPlasma_APC, two muscle, three SuprabasalKer, two Eccrine, five inf_SupraBasal, two BasalKer, two LC_Basal, and two Sebaceous topics, resulting in a final set of 35 topics. Some topics captured mixed cellular signatures reflecting spatial co-localization or coordinated functional programs. In the combined dataset, the Neut_Ker topic co-expressed neutrophil and cytotoxic cell-associated genes (*PRF1*, *ISG15*, *IFI6*, *RSAD2*) alongside inflammatory keratinocyte markers (*S100A2*, *S100A8*), the M2_Th topic co-expressed M2 macrophage markers (*MARCO*, *CLEC10A*) and T helper cell markers (*CD4*, *TRBC2*); and the APC_EarlyPlasma topic co-expressed antigen presentation machinery (*HLA-DRB5*, *HLA-DRB1*) alongside early plasma cell markers (*POU2AF1*, *MZB1*, *JCHAIN*), reflecting spatial co-occurrence.

In the lesion-only dataset, the LC_Basal topic contained Langerhans cell markers (*CD207*, *FCGBP*) alongside basal keratinocyte markers (*TP63*, *DSC3*); the Th1_DC topic co-expressed dendritic cell and myeloid markers (*CXCL9*, *HLA-DQA1*, *CLEC10A*) alongside T helper cell markers (*CD4*, *CD3D*); the Tcm_DC topic co-expressed dendritic cell markers (*CLEC10A*, *CXCL9*) alongside central memory T cell markers (*IL7R*, *TRAC*); the EarlyPlasma_APC topic co-expressed antigen presentation markers (*HLA-DRB5*, *HLA-DRB1*) alongside early plasma cell markers (*POU2AF1*, *MZB1*, *JCHAIN*) similarly reflecting spatial co-occurence; the organised_immune topic co-expressed B cell and germinal centre markers (*CXCL13*, *CD79A*, *POU2AF1*, *CCND2*, *C2*) alongside T cell markers (*TRBC1*, *CCL5*) and stromal fibroblastic reticular cell markers (EMILIN1, MMP1), consistent with TLS-like lymphoid organisation; and the RBC_mac_Tc topic was a low-abundance mixed topic co-expressing red blood cell-associated markers (*HBA2*), macrophage (*FTH1*, *CHI3L1*) alongside cytotoxic T cell markers (*GZMB*, *PRF1*).

### Paired analysis of lesional vs. non-lesional tissue

Topic composition differences between paired lesional and non-lesional samples were assessed using linear mixed-effects models (lme4 v1.1-35, lmerTest v3.1-3) on spot-level deconvolution data (n=5,562 spots, 5 patients, one biopsy per condition per patient). We assumed that each spot represents a transcriptomic observation capturing a unique tissue microenvironment. For each of 23 topics, we fitted: Proportion ~ Condition + (1|Patient_ID), where Condition (lesional vs non-lesional) was a fixed effect and Patient_ID, a random intercept to account for paired samples and patient-specific baselines. Models were fitted using restricted maximum likelihood (REML). Residual spatial autocorrelation between

neighbouring spots within the same biopsy represents a limitation inherent to spot-level spatial transcriptomics analysis.

From each model, we extracted (1): fixed effect coefficient (β) representing mean difference between conditions (2), standard error, t-statistic, degrees of freedom, and p-value, (3) Cohen’s d as standardized effect size (β/√[σ²_patient + σ²_residual]), (4) fold change (lesional/non-lesional mean proportions), and (5) intraclass correlation coefficient (ICC = σ²_patient/[σ²_patient + σ²_residual]) quantifying variance attributable to between-patient differences. P-values were adjusted using Benjamini-Hochberg FDR correction. Cell type associated topics with padj < 0.05 and fold change >1.5 or <0.67 were classified as significantly enriched in lesional or non-lesional tissue, respectively. Effect sizes were interpreted per Cohen’s conventions: |d| < 0.2 (negligible), 0.2-0.5 (small), 0.5-0.8 (medium), >0.8 (large).

### Differential expression analysis

Differential gene expression (DGE) analysis was performed using the Wilcoxon rank-sum test implemented via the FindMarkers function in Seurat (v5.4.0), applied to the SCT assay. PrepSCTFindMarkers function accounted for differences in sequencing depths of the libraries. Genes were included if detected in at least 10% of spots in either group (min.pct=0.1) and with a minimum log2 fold change threshold of 0.5 (logfc.threshold = 0.5). P-values were adjusted using Bonferroni correction.

### Module score calculation

Gene signature module scores for cytotoxicity (*GZMB, PRF1, GNLY, NKG7*) and fibrosis (*COL1A1, COL1A2, COL3A1, COL6A1, COL6A2, MMP2, TIMP2, TIMP3, ACTA2, PDGFRB, SPARC, FBLN1, FBLN2, DCN*) were calculated using Seurat’s AddModuleScore() with default parameters on normalised and scaled counts in SCT assay. To identify regions with the highest cytotoxic activity, spatial spots were stratified based on the cytotoxicity module score distribution. High cytotoxic spots were defined as scores ≥Q3 (top 25%); low cytotoxic spots comprised the remaining 75% (score <Q3).

### Spatial annotation and region classification

Spatial regions were manually annotated using 10x Genomics Loupe Browser based on underlying H&E histology and immune infiltration patterns, prior to and independently of transcriptomic and deconvolution analyses. Annotations were performed by one and reviewed by additional three investigators and any uncertainties were resolved by consensus discussion. For P3, five discrete granulomatous regions were delineated by characteristic histological features (epithelioid histiocyte aggregates) and CD68/CD8A immunofluorescence staining comprising a total of 33 granulomatous spots. Non-granulomatous regions comprised the remaining lesional spots. For P4, regions were further classified into four categories based on morphology and CD68/CD8A immunofluorescence: organized granulomas (180 spots), diffuse immune infiltrate (226 spots), uninvolved stroma (128 spots), and remaining tissue. For P5, epidermal spots were annotated as acanthotic (245 spots from P5_L) or normal (33 spots from P5_NL) based on hematoxylin and eosin staining.

### Enrichment analysis of deconvolved topics

Cell-type-associated topic enrichment was assessed by comparing deconvolved topic proportions between regions or conditions. For pairwise comparisons, two-sided Wilcoxon rank-sum tests were performed (minimum 5 spots per group). For ≥three categories, Kruskal-Wallis tests were followed by pairwise Wilcoxon tests. Fold enrichment was calculated as the ratio of mean proportions between groups. P-values were adjusted using Benjamini-Hochberg correction (padj < 0.05 considered significant).

### Quantification of keratinocyte inflammation across patient samples

The inflamed keratinocyte ratio was calculated per spot as the sum of inflamed keratinocyte topic proportions (inf_BasalKer + inf_SupraBasal + inf_TopKer) divided by the sum of all keratinocyte proportions (inflamed + non-inflamed types). Non-inflamed keratinocyte topics included BasalKer, SuprabasalKer, LC_basal and TopKer.

### Functional annotation and pathway enrichment analysis

DEG lists from each comparison were subjected to functional annotation using Reactome databases using StringDB (STRING 12.5 (59)). Significantly enriched pathways were identified (adjusted p-value < 0.05) and visualized using dot plots as exported from StringDB with increasing signal metric (weighted harmonic mean of observed/expected ratio and -log (FDR). All terms were grouped together with similarity score of 0.8 (based on Jaccard index).

### Subclustering of organized immune regions in P4 and lesional keratinocytes in P5

For subclustering of P4 organized granuloma spots, the 180 spots annotated as organized granulomas were subset from the P4 lesional object. Data were normalized using NormalizeData, and 2,000 variable features were identified using the variance stabilizing transformation (VST) method. Following scaling and PCA (50 PCs), dimensionality was reduced to 10 PCs based on elbow plot inspection. UMAP was computed using dims 1:10, and Louvain clustering was performed using FindNeighbors (dims 1:10) and FindClusters at resolution 1.0, yielding four spatially distinct clusters. Marker genes were identified using FindAllMarkers (min.pct = 0.1, logfc.threshold = 0.5, only.pos = TRUE, padj < 0.05). Clusters were annotated as Plasma_cytotoxic, Fibrotic_core, T_DC_zone, and Lipid_mac based on marker gene expression.

For subclustering of P5 lesional epidermal spots, 245 spots annotated as acanthotic epidermis were subset from the P5 lesional object based on histological annotation (is_epidermis == ‘Epidermis’). Two thousand variable features were identified, data were scaled, and PCA was performed (50 PCs). UMAP and Louvain clustering were performed using dims 1:15 (selected by elbow plot) at resolution 0.8, yielding six epidermal subclusters. Marker genes were identified using FindAllMarkers (min.pct = 0.25, logfc.threshold = 0.5, only.pos = TRUE, padj < 0.05). All subclustering analyses were performed on the SCT assay.

### Immunofluorescence

For IF, 10 μm sections were cut from frozen blocks and mounted on SuperFrost Gold slides (11976299). Sections were fixed in 4% paraformaldehyde for 30 minutes and followed by 0.3% Triton X 100 mediated permeabilization for 15 minutes. Slides were blocked for 1 hour at room temperature with a blocking buffer consisting of 5% normal donkey serum (Sigma-Aldrich, D9663) and 5% normal goat serum (Merck, G9023) prepared in permeabilization buffer. For multiplex IF staining, sections were incubated with primary antibodies for 1 hr: For assessment of tertiary lymphoid structures in P1, sections were stained withCD20 (IGEL/773, NBP2-47840C, Novus Biologicals, 1:100, Dylight-650) and CD3E (CD3-12, ab11089, Abcam, 1:300) to identify B cell and T cell spatial organisation respectively. For assessment of granuloma composition in P3 and P4, sections were stained with CD68 (KP1, ab955, abcam, 1:200) for myeloid cells, and CD8a (C8/144B, 372904, Biolegend, 1:100, AF594) for T cells.

Following primary antibody incubations, sections were washed with PBS, 0.05% BSA (w/v) and incubated with appropriate fluorophore-conjugated secondary antibodies at 1:500 dilution in wash buffer for 30 minutes at room temperature (IgG Donkey anti-rat AF555, ab150154, Abcam for CD3E and CF750 Goat Anti-Mouse IgG, Generon, 20463-250uL for CD68). Nuclei were counterstained with YOYO1 (0.2µM) for 1hr. Specificity of staining was confirmed using secondary antibody-only controls and matched isotype controls for each primary antibody (mouse IgG1 for CD68, mouse IgG2ak for CD20, mouse IgG1k for CD8A, rat IgG for CD3E). Slides were mounted with Prolong Gold. IF images were acquired using a Zeiss, Axio Scan.Z1 slide scanner at 10x. Images were visualized and analysed using ZEN Microscopy Software.

### Statistical testing and visualization

Unless otherwise specified, all tests were two-sided. For regional Wilcoxon/Kruskal-Wallis analyses, tests were performed between spots across patients and remains exploratory in nature, as true population level statistics can only be applied for larger cohort studies.

For multiple pairwise comparisons, Benjamini-Hochberg correction was applied to control FDR. Box plots display median (center horizontal line), interquartile range (box), and 1.5× IQR whiskers. Violin plots show kernel density estimates of the full distribution. In dot plots, size of the dot shows percent expressed in the region/cluster and color of the dot indicates scaled expression. Statistical significance is indicated as: *p < 0.05, **p < 0.01, ***p < 0.001, ****p < 0.0001.

All analyses were performed in R (version 4.5.0) using Seurat (version Seurat_5.4.0), STdeconvolve (version 1.3.2), SCpubr (version 3.0.0), ComplexHeatmap (version 2.24.1), Scatterpie (v0.2.6), ggpubr (v0.6.0) and ggplot2 (version 3.5.2) packages.

## Data Availability

The sequencing data have been deposited in GEO (GSE317987). The processed data are available as Rds files on Zenodo: https://doi.org/10.5281/zenodo.20554918. Analysis code is available at https://github.com/NidhiSDey/SpatialCL.git.
