# Supplementary material for: Spatial mapping of Ethiopian cutaneous leishmaniasis lesions reveals distinct tissue level immune programs
Source: Front Immunol. 2026 Jul 21;17:1870524. doi: 10.3389/fimmu.2026.1870524 (PMC13433223; doi:10.3389/fimmu.2026.1870524)
Supplement: Supplementary file 3 [file DataSheet3.zip › Data Sheet 3/Supplementary Table 1.pdf]

Table 1. Sociodemographic data of patients

Age (years)*	21.5 (20.3-25)
Sex (Male:Female)	6:00
Occupation	
Student	2/6 (33.3%)
Farmer	2/6 (33.3%)
Clerk	1/6 (16.7%)
Unemployed	1/6 (16.7%)
Level of education	
Primary school	1/6 (16.7%)
High school	4/6 (66.7%)
None	1/6 (16.7%)

*values given in Median (Interquartile Range)
