# Supplementary material for: Spatial mapping of Ethiopian cutaneous leishmaniasis lesions reveals distinct tissue level immune programs
Source: Front Immunol. 2026 Jul 21;17:1870524. doi: 10.3389/fimmu.2026.1870524 (PMC13433223; doi:10.3389/fimmu.2026.1870524)
Supplement: Supplementary file 3 [file DataSheet3.zip › Data Sheet 3/Supplementary Table 2.pdf]

Supplementary Table 2: Lesion assessment at follow-up visits

PID	Treatment cycles	Treatment	Previous history of treatment at enrollment	First treatment duration in days (after enrollment)	Second treatment duration in days (after enrollment)	Overall treatment response at D28	Number of active lesions (active/total lesions) at D28	Lesion size index lesion (cm2) at D28	Characterization of index lesion at D28	Characterization of other active lesion at D28	Overall treatment response at M6
P1	2	SSG	No	28	15	Substantial improvement (50-99%)	1/1	2	crusted, macrochelial, swollen, erythematous, super-infected	NA	Cure (100%)
P2	1	SSG	No	28	NA	Substantial improvement (50-99%)	1/1	6	plaque, scaly, crusted, dry, hyperpigmented	NA	Cure (100%)
P3	2	SSG	Herbal treatment 1 month before enrollment	30	30	Substantial improvement (50-99%)	3/3	2	plaque, scaly, dry	ulcerated, scaly, hyperpigmented	LTFU
P4	1	SSG	No	30	NA	Substantial improvement (50-99%)	1/1	8	plaque, scaly, dry, exfoliative	NA	LTFU
P5	1	SSG	No	28	NA	Substantial improvement (50-99%)	1/3	12	plaque, crusted, dry	NA	Cure (100%)

D28 = Day 28, M6 = Month 6, NA = not applicable, LTFU = lost to follow up, SSG= Sodium Stibogluconate
D28 (window: +5d vs -5d)
M6 (+/-2M)
